# Remote Digital Psychiatry for Mobile Mental Health Assessment and Therapy: MindLogger Platform Development Study

**DOI:** 10.2196/22369

**Published:** 2021-11-11

**Authors:** Arno Klein, Jon Clucas, Anirudh Krishnakumar, Satrajit S Ghosh, Wilhelm Van Auken, Benjamin Thonet, Ihor Sabram, Nino Acuna, Anisha Keshavan, Henry Rossiter, Yao Xiao, Sergey Semenuta, Alessandra Badioli, Kseniia Konishcheva, Sanu Ann Abraham, Lindsay M Alexander, Kathleen R Merikangas, Joel Swendsen, Ariel B Lindner, Michael P Milham

**Affiliations:** 1 MATTER Lab Child Mind Institute New York, NY United States; 2 Computational Neuroimaging Lab Child Mind Institute New York, NY United States; 3 Université de Paris and INSERM U1284 SEED unit Centre for Research and Interdisciplinarity (CRI) Paris France; 4 ETH Library Lab ETH Zurich and Citizen Science Centre Zurich Switzerland; 5 McGovern Institute for Brain Research Massachusetts Institute of Technology Cambridge, MA United States; 6 Department of Otolaryngology - Head and Neck Surgery Harvard Medical School Cambridge, MA United States; 7 Octave Bioscience Menlo Park, CA United States; 8 Computational Engineering University of Texas at Austin Austin, TX United States; 9 Center for the Developing Brain Child Mind Institute New York, NY United States; 10 National Institute of Mental Health Bethesda, MD United States; 11 National Center for Scientific Research University of Bordeaux EPHE PSL University Bordeaux France; 12 Center for Biomedical Imaging and Neuromodulation Nathan S. Kline Institute for Psychiatric Research Orangeburg, NY United States

**Keywords:** mental health, mHealth, mobile health, digital health, eHealth, digital psychiatry, digital phenotyping, teletherapy, mobile device, mobile phone, smartphone, ecological momentary assessment, ecological momentary intervention, EMA, EMI, ESM, experience sampling, experience sampling methods

## Abstract

**Background:**

Universal access to assessment and treatment of mental health and learning disorders remains a significant and unmet need. There are many people without access to care because of economic, geographic, and cultural barriers, as well as the limited availability of clinical experts who could help advance our understanding and treatment of mental health.

**Objective:**

This study aims to create an open, configurable software platform to build clinical measures, mobile assessments, tasks, and interventions without programming expertise. Specifically, our primary requirements include an administrator interface for creating and scheduling recurring and customized questionnaires where end users receive and respond to scheduled notifications via an iOS or Android app on a mobile device. Such a platform would help relieve overwhelmed health systems and empower remote and disadvantaged subgroups in need of accurate and effective information, assessment, and care. This platform has the potential to advance scientific research by supporting the collection of data with instruments tailored to specific scientific questions from large, distributed, and diverse populations.

**Methods:**

We searched for products that satisfy these requirements. We designed and developed a new software platform called *MindLogger*, which exceeds the requirements. To demonstrate the platform’s configurability, we built multiple *applets* (collections of activities) within the MindLogger mobile app and deployed several of them, including a comprehensive set of assessments underway in a large-scale, longitudinal mental health study.

**Results:**

Of the hundreds of products we researched, we found 10 that met our primary requirements with 4 that support end-to-end encryption, 2 that enable restricted access to individual users’ data, 1 that provides open-source software, and none that satisfy all three. We compared features related to information presentation and data capture capabilities; privacy and security; and access to the product, code, and data. We successfully built MindLogger mobile and web applications, as well as web browser–based tools for building and editing new applets and for administering them to end users. MindLogger has end-to-end encryption, enables restricted access, is open source, and supports a variety of data collection features. One applet is currently collecting data from children and adolescents in our mental health study, and other applets are in different stages of testing and deployment for use in clinical and research settings.

**Conclusions:**

We demonstrated the flexibility and applicability of the MindLogger platform through its deployment in a large-scale, longitudinal, mobile mental health study and by building a variety of other mental health–related applets. With this release, we encourage a broad range of users to apply the MindLogger platform to create and test applets to advance health care and scientific research. We hope that increasing the availability of applets designed to assess and administer interventions will facilitate access to health care in the general population.

## Introduction

### Background

This section begins with acknowledging the global burden of mental illness and barriers to care, as well as the increasing need of patients, clinicians, and scientists for digital, mobile, remote mental health care and research. We have then highlighted the advantages of mobile apps, passive monitoring, and experience sampling. To provide context for the rest of the paper, we have described 2 studies: the Healthy Brain Network study and the National Institute of Mental Health (NIMH) Family Study of Affective Spectrum Disorders.

### Global Burden of Mental Illness and Barriers to Care

The global burden of mental illnesses is staggering. Epidemiologic studies indicate that 75% of all diagnosable psychiatric disorders begin before the age of 24 years [1] and the lifetime prevalence of a severe disorder among children and adolescents is 21.4%. The most common diagnoses during childhood are anxiety disorders, attention-deficit/hyperactivity disorder, and mood disorders [2]. Despite their high prevalence, only about 50% of children with a mental health disorder receive treatment (Centers for Disease Control and Prevention, NIMH). Adults and children remain untreated, even though effective treatments exist. The World Health Organization has reported large median treatment gaps for alcohol abuse and dependence (78%), generalized anxiety disorder (58%), obsessive-compulsive disorder (57%), depression (56%), dysthymia (56%), panic disorder (56%), bipolar disorder (50%), and schizophrenia (32%) [3,4]. There is a general dearth of mental health resources, especially in low- to middle-income settings [5] and for disadvantaged youth [6], and there are many barriers to care that do not involve finances, insurance, and availability of treatments [6-16]. Simply scheduling an appointment in the United States can be difficult [8], and wait times may take months [8,11]. Additional barriers include concerns about stigma and privacy, as well as exceptional circumstances when telehealth is the only option, such as with the current COVID-19 crisis.

### An Increasing Need for Digital, Mobile, Remote Mental Health Care, and Research

People seeking mental health care can use web-based resources to overcome barriers to treatment, such as distance, cost, long waits, inconvenience, stigma, privacy, and problems associated with in-person visits. In recent years, people have been increasingly turning to the internet as a preferred source of knowledge about mental health [7,17,18]. However, the abundance of inaccurate and misleading information on the internet makes it difficult to evaluate the credibility of information sources, the veracity of claims, and the effectiveness of recommendations and therapies. In addition, there are many scenarios in which obtaining data or care without access to the internet or a computer is important. Furthermore, receiving scheduled notifications at any time and place can be critical for a realistic assessment and effective intervention. In the context of these requirements, mobile devices with dedicated apps offer a better way of receiving vetted, curated content and of communicating relevant information without having to sit in front of a computer or find the right person to talk to at the right time.

Clinicians and health organizations, particularly those in the mental health sector, are overwhelmed. General health practitioners, in particular, struggle to meet the needs of their local communities. Better assistance is required to identify mental health issues during checkups to ensure that these concerns are caught early on and that patients benefit from referral to a specialist. Health organizations struggle to provide broader assistance to the vast majority of people around the world who have limited or no access to mental health resources. They need better tools to scale up mental health efforts that reduce or remove face-to-face interactions to inform, assess, and provide therapy. Digital and mobile tools are especially important to reach people who need care and who are in remote areas, in socioeconomically disadvantaged conditions, or in situations where there is profound stigma surrounding mental illness. Telehealth options that involve video teleconferencing with a health care provider are becoming more prominent and have some evidence of scalability [19]. However, these services require the time and attention of expensive and limited human resources, and therefore do not scale as well as digital, mobile, and remote resources that do not require human interaction. The current COVID-19 crisis has made it starkly apparent that telehealth, with or without human interaction, may at times be the only option for seeking and receiving care. This pandemic is causing a widespread impact on mental health, from anxiety and stress related to helplessness, fear, and uncertainties of the crisis itself to the isolation and loneliness of home confinement and social distancing [20,21]. The need for remotely administered information, assessment, and therapy will only grow as nations have been forced to discover the extensive possibilities of these novel technologies.

Scientists often struggle to acquire relevant mental health and behavioral data when using traditional research paradigms. Study participant recruitment efforts are usually restricted to individuals who can be evaluated in person and provide written consent, which drastically reduces the size and scope of study samples. Smaller sample sizes are less likely to detect effects that exist at the population level, so for data to be relevant, robust, and replicable, it is important to frequently sample from large and diverse populations. Off-the-shelf, web-based tools, such as mobile apps for collecting data, do not meet the needs of most scientists who must configure their data collection tools to reach the right population and address specific questions of scientific and clinical relevance. Even tools that are configurable often require considerable mobile app software engineering expertise that is outside of most laboratories’ capabilities. For laboratories attempting to create their own mobile app from scratch, they may turn to outsourcing for its development only to find that it is far more costly than anticipated and requires consistent maintenance and upgrading to keep up with changing software dependencies, operating system versions, and hardware, let alone the changing requirements of the research itself. In addition to the engineering, content, design, and financial challenges of creating mobile apps, there can be considerable logistical and governance challenges involved in dissemination and the incorporation of an electronic consent process.

Appropriately constructed, configured, and vetted mobile apps are currently the most promising avenue for satisfying all of the abovementioned requirements, as mobile devices with internet access are rapidly becoming widely available to diverse populations around the world. It is currently estimated that 60% of the world’s population uses the internet and that 93% of users gain internet access through mobile devices [22]. They provide a convenient way to passively or actively collect and present information that can be relevant to the natural variations and social and environmental contexts of people’s lives. Passive methods usually involve sensors carried or worn on some part of the body and can collect objective, real-world data about participants to monitor motor activity, sleep, heart rate, cognition, behaviors, mood, and physiological states, as well as detect important outcomes such as medication response [23]. Some recent examples of sensor-based technologies that track mental health include phone apps, such as Northwestern University’s Center for Behavioral Intervention Technologies’ Purple [24,25] and Intellicare [26,27] platforms, Harvard University’s Beiwe platform [28,29], and apps such as mPower [30,31] built on top of Apple’s ResearchKit for iOS [32] and ResearchStack for Android [33]. The Beth Israel Deaconess Medical Center maintains an extensive database of apps that claim to respond to mental health needs [34] and provides a protocol for their evaluation [35,36]. There is an unmet need for a free, open, configurable platform to translate pencil-and-paper assessment instruments, cognitive tasks, and therapies into attractive, engaging, and effective digital, mobile, and remote tools that exceed current standards of privacy, security, and accessibility.

One important limitation in the interpretation of data acquired through passive monitoring is the lack of contextual information on variables that may influence sleep, activity, or mood changes inferred from speech, texting, GPS location, or other interactions with mobile devices. Although studies have combined subjective symptom ratings with passive monitoring, retrospective reporting over the past week or month is common among many traditional clinical questionnaires and may miss important real-time associations between subjective and objective data. Gaining insight into the directional associations between events and psychological states and their association with sleep and physical activity can be enhanced through the administration of tools that simultaneously capture descriptions of symptoms of mood, cognition, and other subjective experiences, which remain the core components of psychiatric disorder criteria. These active methods of recording individuals’ internal states and experiences include explicit self-reports that may range from occasional and detailed survey instruments to more frequent, brief, and in-the-moment questionnaires. Repeated assessment of people in real time is best known as *experience sampling* [37] and in the context of medicine and physiological or event-related data capture has been referred to as ecological momentary assessment (EMA) [38]. As pointed out by Marije aan het Rot et al [39] in their excellent review of experience sampling methods and EMA [39], the 2 terms are increasingly used together, so we will simply refer to them as *experience sampling*.

As described by Trull and Ebner-Priemer [40], experience sampling offers major benefits over traditional clinical assessments, including the reduction of retrospective bias, real-time tracking of dynamic processes, simultaneous integration of multi-level data (eg, biological and psychological), characterization of context-specific relationships of behaviors and symptoms, inclusion of feedback, and enhanced generalizability of results. Experience sampling has been shown to be highly feasible and valid in the assessment of diverse categories of mental illness, including patients with mood disorders, anxiety disorders, substance use disorders, and psychosis [41-45] as well as in the assessment of transdiagnostic mental health issues such as suicidal ideation [46]. More recently, experience sampling has been used to assess cognitive functions [47]. Batteries of cognitive tasks, such as the NIH Toolbox [48,49], ACE [50], and Cambridge Cognition [51], are primarily used for research and are not currently intended for clinical practice; they are for the most part commercial, proprietary, and permit limited (if any) configuration options for presenting and collecting data. Although mental health professionals may not adopt new technologies for clinical assessment more readily than traditional tools [52], their use in clinical practice may be encouraged by increasing their personalization and integration into standard care [53].

### The Healthy Brain Network Study and NIMH Family Study of Affective Spectrum Disorders

The Child Mind Institute’s Healthy Brain Network study [54] is an ongoing initiative focused on creating and sharing a biobank of data from 10,000 New York area participants (age group 5-21 years). The biobank houses data on psychiatric, behavioral, cognitive, and lifestyle phenotypes, as well as multimodal brain imaging (resting and naturalistic viewing functional magnetic resonance imaging (MRI), diffusion MRI, and morphometric MRI), electroencephalography, eye tracking, voice and video recording, genetics, and actigraphy.

The NIMH Family Study of Affective Spectrum Disorders was a large, community-based, controlled family study [55] that collected assessment data 4 times per day for 2 weeks from phones provided to participants of the study. The assessments included questions about daily life experiences and behaviors at the moment of acquisition (current location, social company, performance of specific behaviors, and mood states) and since the previous assessment or since waking up (experience of daily events and event negativity, food intake, substance use, experience of headache, and specific symptoms). Assessments at the beginning of the day also included questions about duration, quality, and problems with sleep, and at the end of the day included ratings of the stressfulness of the day, food craving for the day, and specific physical symptoms (gastrointestinal symptoms and muscle pain). Response options included Likert scales for dimensional constructs (such as mood or event negativity) and checklists for multiple responses (such as for food consumed) or single responses (such as current physical location).

The data from the NIMH Family Study that evaluated the association between daily events and emotional experience yielded important differences in patterns of reactivity among the major subtypes of mood disorders, including bipolar I disorder, bipolar II disorder, major depression, anxiety disorders without a mood disorder, and controls [56]. These findings demonstrate how experience sampling is a particularly well-adapted tool for assessing affective dynamics as well as emotional reactivity following daily life events. The value of combined passive and active monitoring in this study further showed bidirectional associations between energy, motor activity, and sleep, and unidirectional associations between activity and mood, suggesting that increased activity could be used as an intervention for depression [57]. Using the novel analytic approach of fragmentation to test the stability and instability of emotional states in this study showed greater instability of energy and attention in people with a history of bipolar I disorder, whereas those with bipolar II disorder or major depression exhibited greater fragmentation of mood and anxiety [58].

### Outline of the Paper

In this paper, we (1) review customizable, mobile, experience sampling products for configurable data collection and content delivery, (2) summarize the motivation for and development of a new mobile platform called MindLogger, and (3) describe MindLogger applets, including an initial use case that applies a MindLogger version of the NIMH Family Study’s app as part of the Healthy Brain Network study.

## Methods

### Overview

In this section, we have discussed our criteria for selecting and evaluating customizable, mobile, experience sampling products; provided an overview of the development of the MindLogger platform for experience sampling and interventions; and presented an example app of MindLogger in a large-scale mental health study. The *Results* section follows up on each of these, presenting the results of the product review, the current state of the MindLogger platform, and example apps of MindLogger.

### Criteria for Reviewing Customizable, Mobile, and Experience Sampling Products

In this review, we did not consider mobile apps limited to specific assessments, cognitive tasks, or therapies, but rather examined platforms that enable the creation and distribution of such apps. We wanted to find products that have an administrator interface for creating and scheduling recurring, customized questionnaires, where users receive and respond to scheduled notifications on a mobile device.

The detailed outline of the protocol we followed has been provided in Multimedia Appendix 1, which is briefly summarized in the paper. Over the last 3 years, clinical and research collaborators and colleagues have helped us gather information about products with desired characteristics. To extend this search, we conducted 3 queries in Google’s search engine (without quotation marks or Boolean operators): (1) *digital electronic data capture systems*, to broadly identify any electronic tools for capturing data; (2) *mobile phone software sensor data collection*, to identify mobile data collection software that may involve sensors; and (3) *alternative to Qualtrics*, to identify alternatives to one of the most prominent products that enables web-based customization of surveys (although Qualtrics itself does not currently have a mobile app). We visited the websites of the first 20 search results from each query (not including advertisements) and identified the candidate products. For example, if a website listed the *Top Ten Apps for...*, we would include these 10 apps in our initial set of candidates. We then filtered this set using the following inclusion criteria: the candidate product had to (1) be in current use, (2) have (Android and iOS) mobile apps, and (3) have an administrative user interface for creating and scheduling times and days for recurring, customized questionnaires, where end users receive and respond to scheduled notifications via an iOS or Android app on a mobile device. Where there was any ambiguity, we contacted the company or organization to clarify, scheduled a web-based demonstration, and requested a free trial to explore the product. We excluded products that do not currently fulfill the above requirements or for which we could not receive a demonstration or trial without a legal agreement. We also excluded products that require SMS text messaging, email, or other modes of communication outside of their mobile app to send and receive notifications.

Because the type of notification is important in experience sampling applications, we identified which products can deliver local operating system notifications or push notifications, where an end user receives notifications in their mobile device’s notification bar at scheduled times, and a tap on a notification takes them to their scheduled activity within the mobile app. Local operating system notifications do not require an internet connection at the time that the notification is to be received, whereas push notifications do, and both of these are distinct from simple in-app notifications, which require the end user to use the app to see their notifications.

We collected additional information from product websites, and via teleconferences and web-based demonstrations with the product creators, and from free trials to determine the degree to which each is (1) customizable in its information presentation and data capture capabilities, (2) private and secure, and (3) accessible (easy to use, economical, and open source). Customization is important to ensure that content, language, and presentation can be adapted and updated to meet the specific needs of a given population of intended end users, and to expand the scope of possible dimensions to assess, analyses to perform, and inferences to make. Privacy and security is a central concern, especially as we conduct research involving a triply vulnerable population of end users: (1) child and adolescent (2) patients with (3) mental health and learning disorders. Data access, encryption, and deletion capabilities are the primary considerations in this domain. *Accessible* can mean many things; here we refer to whether and how the administrator can access the product, its source code, or its data. The degree to which a product is affordable will often determine the degree to which it is adopted. Possibly, the most stringent accessibility criterion one could have is for the software to be open source. Open-source software is important because the product does not live or die with a given company, provides an opportunity for anyone to build on and improve the software, and it is open to greater scrutiny to ensure the quality of the software, accuracy of any claims made about the software, and transparency of clinical and scientific practices that use the software. Each of these characteristics offers a competitive advantage; however, they should be considered together. For example, a company offering a free product can store or transmit user data insecurely.

### Development of the MindLogger Platform

MindLogger [59] is intended as an *open ecosystem to create, edit, share, and administer mobile or web applets for data collection and content delivery*. We use the term *applet* to refer to a customized collection of activities within the MindLogger app administered to target end users. Our focus with MindLogger is to easily create and edit digital mental health assessments and interventions and administer them to users via mobile or web applets. The 3 key innovations we sought to accomplish with MindLogger were *customizability* of content, response options, and appearance; an extensive library of applets built using *open standards* (open, reusable parts defined by an open protocol), and distribution as a *single app* that appears differently to different user groups.

MindLogger’s development began in 2017, with a focus on mental health assessments. After the significant development of an early prototype for mental health research and clinical colleagues at the Child Mind Institute and the Child Mind Medical Practice in New York City, we revisited development with a greater emphasis on human-centered design [60,61]. We included a variety of key stakeholders to ensure that their needs would be met by the platform: clinicians (psychiatrists, psychologists, social workers, etc), scientists (neuroscientists, cognitive psychologists, etc), and directors of schools specializing in learning and developmental disorders, as well as technology consultants. We integrated their feedback at different stages throughout the development of the platform. Regarding user experience, both structured and informal user feedback collection has been iterative and ongoing throughout the development process. The MindLogger apps described in this paper use an assessment frequency, duration, and question content that has been substantially validated over recent years by members of our team for a wide range of age groups (children, adults, and older adults) [62-66], for healthy individuals [62,63,67], and for persons with diverse forms of mental [62,63,67-71] or physical [72-74] disorder. The NIMH’s predecessor to the MindLogger applet described below (using a highly similar protocol for daily assessments and with identical question content) documented an average completion rate of 77.9% (SE 0.81%) for repeated daily assessments, with no significant fatigue effect, defined as an increase in missing data as a function of time in the study (ordinary least squares linear model β coefficient=–.041; *P*<.001). In addition to user experience considerations and other stakeholder feedback, the information we gathered over the last 3 years that preceded the above review of customizable, mobile, experience sampling products also helped to guide MindLogger development.

### Application of MindLogger in the Healthy Brain Network Study

To obtain more in-depth information on real-time tracking of emotions, behavior, daily activities, and their contextual influences in the Healthy Brain Network study [54], we adapted the combined actigraphy and experience sampling mobile assessment tools and content from the NIMH Family Study of Affective Spectrum Disorders [55]. Although the findings of that study (summarized in the Introduction section) were primarily based on adult samples, the inclusion of a substantial subset of offspring aged 10-18 years of parents with mood disorders and controls provided compelling evidence for the feasibility, acceptability, and clinical significance of experience sampling in youth. Therefore, the goal of the present initiative was to create a version in MindLogger, with updated content (particularly with regard to sleep, positive and negative thoughts, food and drink, internet, and social media), enhanced with clarification of the content, inclusion of colorful images, and formats adapted for children and young adults to encourage engagement [75].

## Results

### Overview

In this section, we present the results of our review of customizable, mobile, experience sampling products, the current state of the MindLogger platform (including roles and permissions, software architecture, and current set of features), and the applet we have deployed in the Healthy Brain Network study.

### Results of the Review of Customizable, Mobile, and Experience Sampling Products

**Figure 1:**
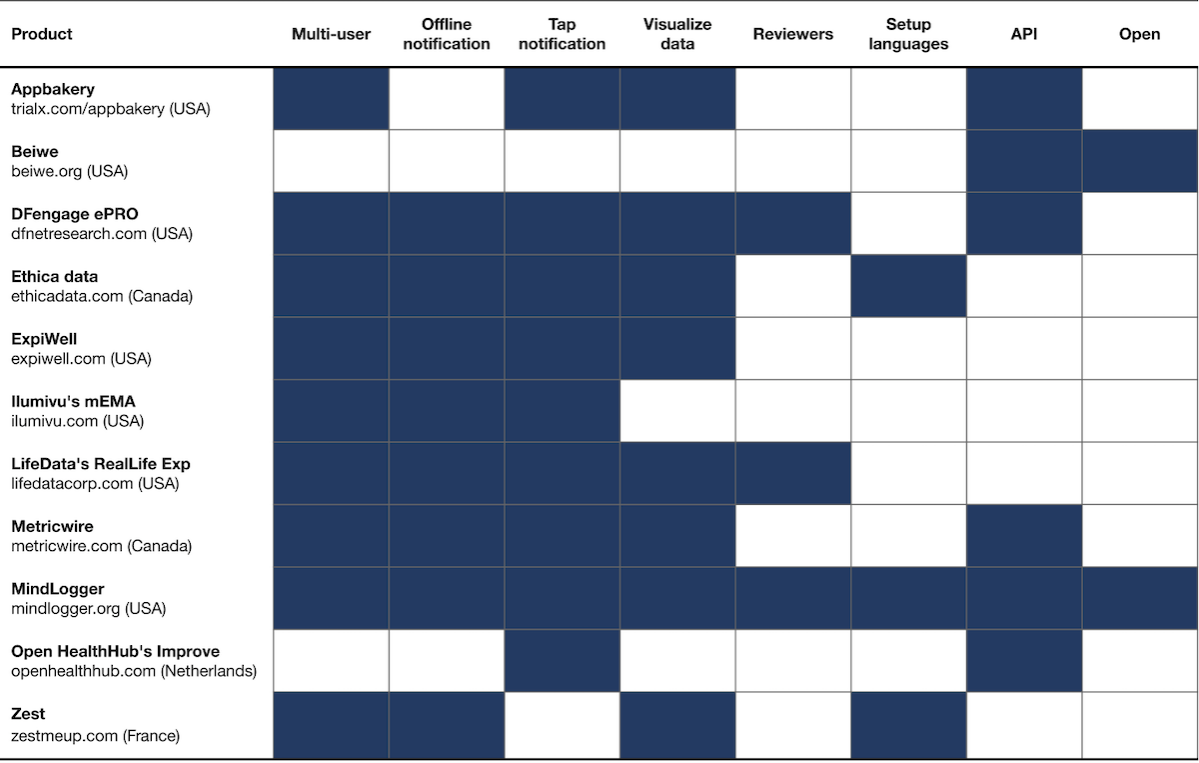
Access to experience sampling products, software, and data (filled means yes). API: application programmer interface.

**Figure 2:**
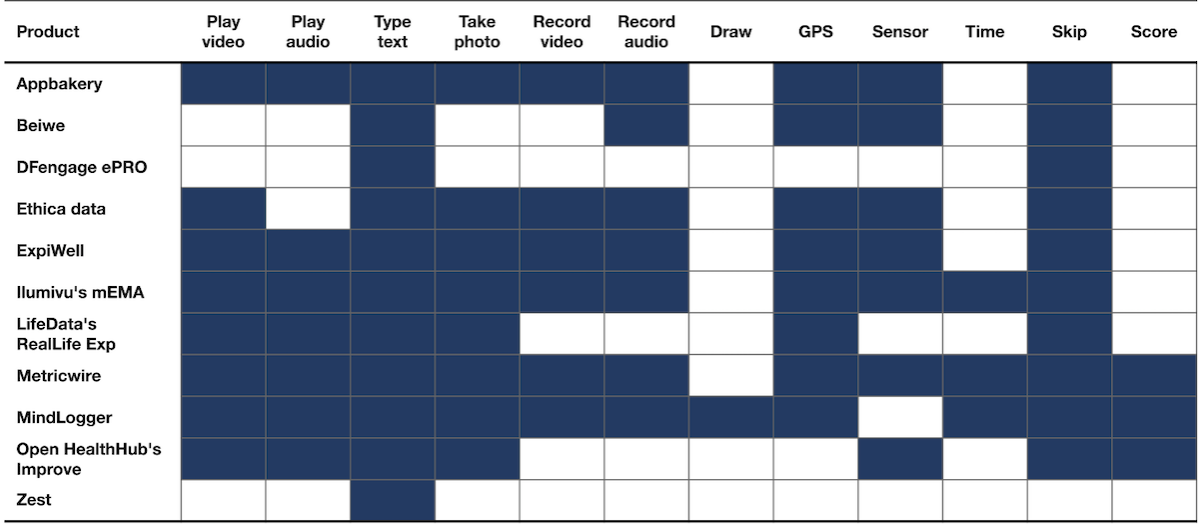
Presentation and data capture features of experience sampling products (filled means yes).

**Figure 3:**
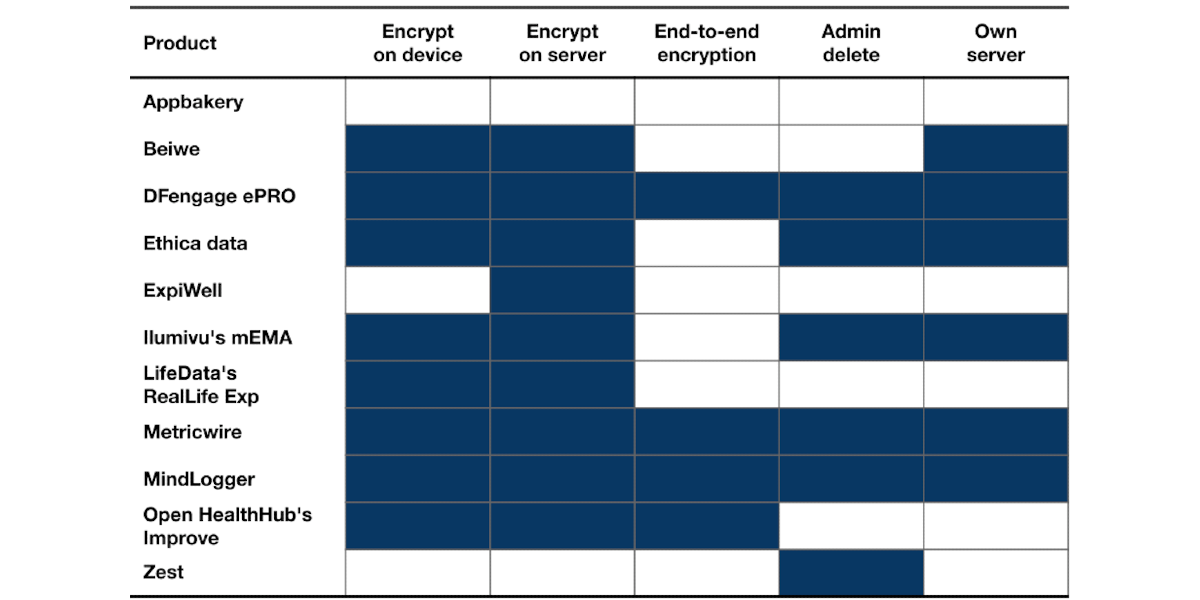
Privacy and security of experience sampling products (filled means yes).

Our search resulted in 392 products, of which 315 appear to be in current use. Multimedia Appendix 1 contains a list of 101 products that have Android and/or iOS mobile apps. Upon closer inspection of their websites, 59 appeared relevant to scheduling questionnaires and notifications for a group of respondents, so we contacted the 59 products’ companies/organizations through their web-based contact forms or via email to clarify their products’ capabilities and exchanged emails with the 47 companies that responded. On the basis of these exchanges, we were able to identify 21 products that appeared to satisfy our primary criteria (administrator interface for creating and scheduling recurring, customized questionnaires, where users receive and respond to scheduled notifications on a mobile device). Five potential candidates were omitted as they are intended for use by internal business employees of a company, not by patients or by participants of a study, and require individual licenses, log-ins, or fees per device. Two more candidates were omitted as they required a legal agreement to demonstrate their products. Of the 14 remaining products for which we engaged in demonstrations and free trials, 10 met the primary criteria and are included in Figures 1-3. In these figures, *yes* or *no* (filled vs empty) correspond to the questions in the bulleted lists below. The questions represent specific instances of broader criteria that can be complex and nuanced.

- *Multiuser*: Can more than one end user access the app on the same mobile device, even if it means logging out and logging back in again?
- *Offline notification*: Can end users receive and respond to notifications without an internet connection?
- *Tap notification*: When an end user taps on a notification in their mobile device’s notification bar, does it take them directly to their scheduled activity within the app?
- *Visualize data*: Is there a data visualization dashboard to review any individual end user’s response data?
- *Reviewers*: If there is a data visualization dashboard, can an administrator give someone access to review only one end user’s response data in the dashboard?
- *Setup languages*: When administrators create a customized questionnaire, can they choose from at least 5 different languages to use the interface, in addition to English? (This is distinct from how many different languages the end users can see.)
- *API*: Is there a consumer-facing application programming interface (API)?
- *Open*: Is the product’s mobile app software fully open source and free to set up?

Figure 1 contains information about access to the product, software, and data (for both Android and iOS).

Figure 2 contains information about presentation and data capture features:

- *Play video/audio*: When administrators create an activity, can they include video or audio clips?
- *Type text, take photo, record video/audio, draw*: When administrators create an activity, can they include any of the following to capture data from end users? (Text entry, camera photo, audio/video recording, and drawing.)
- *GPS*: Can GPS location data be acquired through the app?
- *Sensor*: Can any additional sensor (eg, accelerometer) data be acquired?
- *Time*: Can any question include a countdown or timer?
- *Logic*: Can the response to a question determine which is the follow-up question (skip/branch logic)?
- *Score*: Can a questionnaire’s scoring logic be entered when creating the questionnaire?

Figure 3 contains information about privacy and security:

- *Encrypt on (device, server)*: Does the product encrypt data on the mobile device or on the server?
- *End-to-end encryption*: Is the product end-to-end encrypted or could someone within the company or organization hosting apps on their server see (even anonymized respondents’) response data?
- *Admin delete*: Can an administrator delete an individual end user’s data without having to make a request from the product creator?
- *Own server*: Can the product be hosted on an administrator’s own server?

### Current State of the MindLogger Platform

#### Roles and Permissions

**Figure 4:**
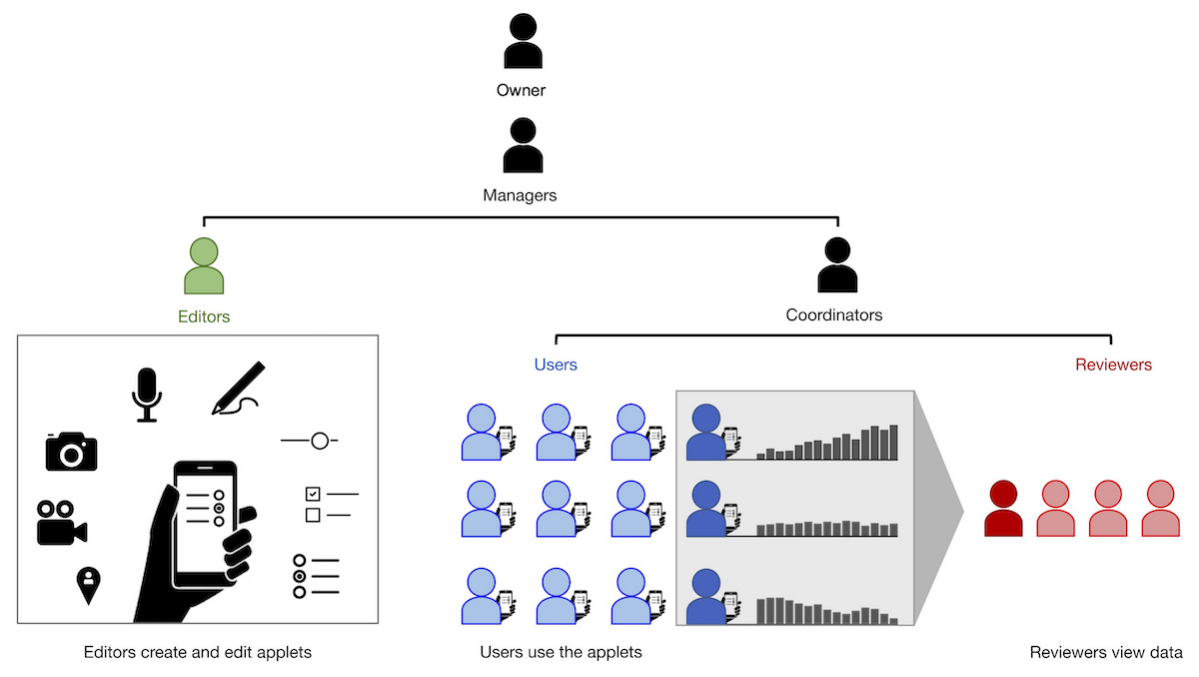
MindLogger schematic. Left: select, edit, or create activities with scheduled notifications, such as questionnaires, tasks, or interventions, for use on mobile (iOS, Android) devices or the web. Middle: assign yourself or others to do these activities for data collection, annotation, research, or remote clinical assessment or therapy. Right: view end user data for which you have access.

**Figure 5:**
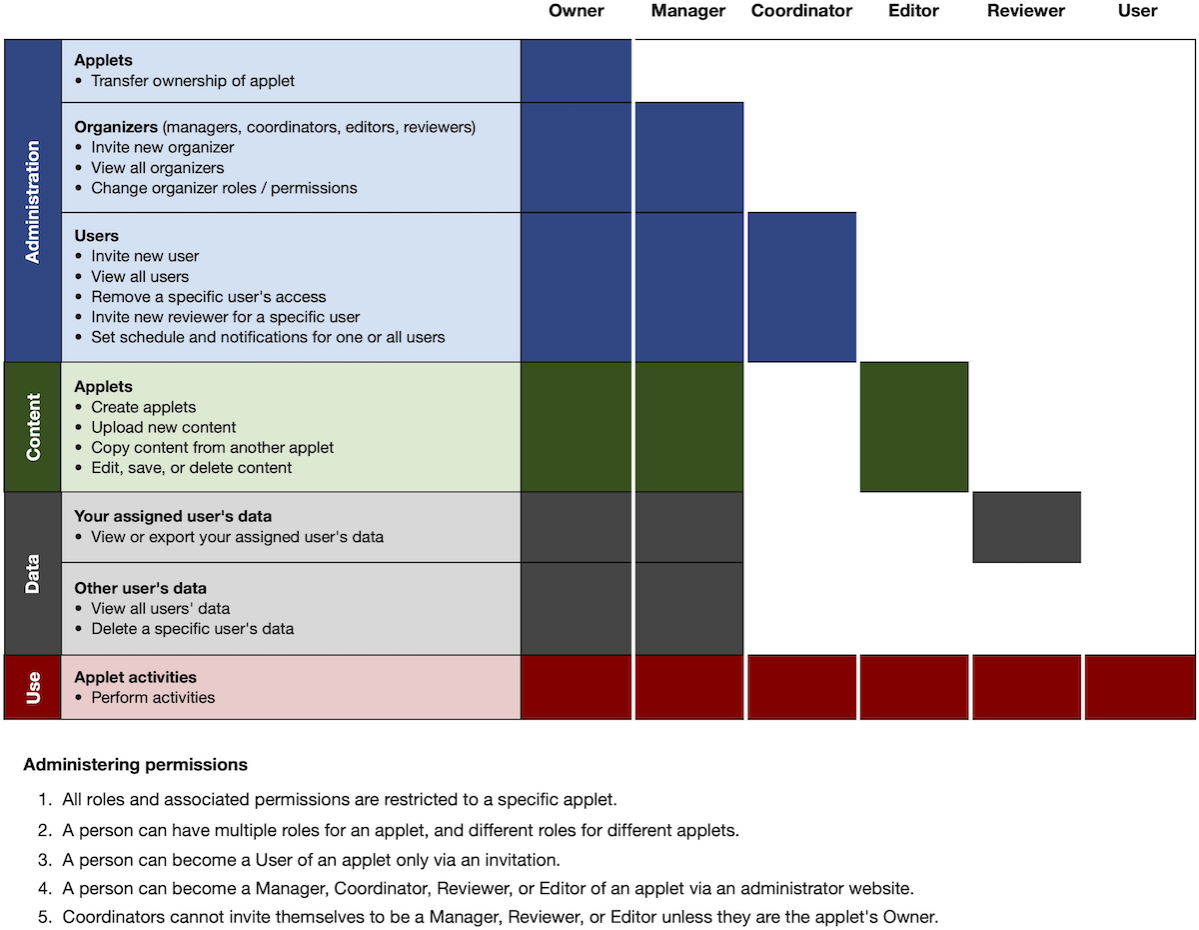
MindLogger roles and permissions for administration, content, use, and data management.

Figure 4 shows a schematic of MindLogger, where an administrator selects, edits, or creates an applet; administers the applet to end users; and views or makes use of their data. Figure 5 outlines the different roles (owner, manager, coordinator, editor, user, or reviewer) and their permissions with regard to administration, content, use, and data management. For instance, a principal investigator of a research study could be the owner of a new applet as well as its editor and might assign a laboratory manager who recruits participants (applet users) to be the manager of the applet, and 2 data analysts to be the reviewers of all deidentified participants’ data. As a second example, a clinical director of a pediatric mental health clinic could be the owner of a copy of an applet containing assessments, assigns a clinical coordinator who manages patients (applet users) to be a manager of the applet, and assigns parents to be reviewers of their child’s responses. As a trivial example, anyone could sign up for a MindLogger account and own their own applet and be its only user. This last scenario enables any user to customize their own applet to send scheduled notifications to themselves to remind themselves to take their medication, perform breathing exercises, practice mindfulness meditation, or log their thoughts within the app.

#### MindLogger Software Architecture

**Figure 6:**
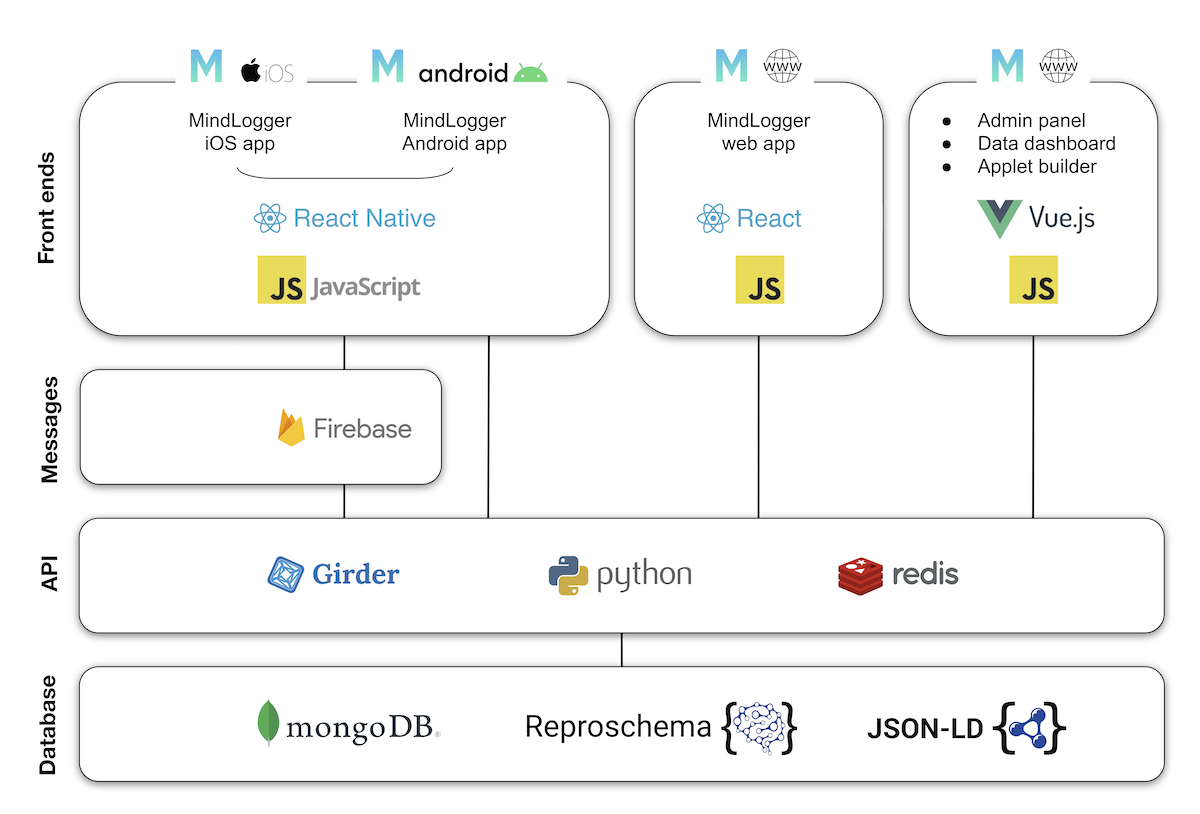
MindLogger software architecture diagram. API: application programmer interface.

MindLogger's software architecture (Figure 6) consists of a set of end user–facing front ends (2 mobile apps and a web application) and organizer-facing front ends (an admin panel, data dashboard, and applet builder) with a shared RESTful HTTP API using MongoDB [76] for data storage. The mobile front ends, Android, and iOS apps are built using React Native [77]. This allows us to share a single code base across mobile platforms, resulting in increased speed of development and ease, and cost-effectiveness of maintenance. The web application is a ReactJS browser-based counterpart to mobile apps and currently provides a subset of their functionality. Administrators (managers, coordinators, editors, and reviewers) have access to different single-page applications built using VueJS [78]. The admin panel and applet builder manage user roles and applets, and the data dashboard is used to review user data, with custom charts implemented using d3.js [79]. The computer security firm Alpine Security [80] conducted extensive cybersecurity black, gray, and white penetration tests to ensure that MindLogger follows best practices for privacy and security. These practices can be adapted to the specific regulations and guidelines of different countries, including the General Data Protection Regulations of the European Union [81], which are among the strictest concerning data use, access, and storage. As shown in Figure 3, MindLogger has end-to-end encryption, permits administrators to delete an individual’s data, and can be set up on one’s own server (accommodating European regulations concerning the physical location of data processing and storage [81]), so the platform should already meet the security requirements of most use cases.

Our application front-end and backend code base is accessible as web-based GitHub repositories [59] and is licensed under an extremely permissive Open Source Initiative–approved open-source license, the Common Public Attribution License (CPAL-1.0) [82]. The license requires that attribution be given by including (1) the copyright notice: “Copyright (c) 2017 MATTER Lab at the Child Mind Institute,” (2) the MATTER Lab website address, (3) the Child Mind Institute’s logo, and (4) the attribution phrase: “Child Mind Institute product intended for building applications for good.” We include the attribution phrase to give credit to the developers while also making it clear that although we intend for people to build MindLogger applets that will be benevolent, we have no control over their intent, content, or the data they collect [83].

Our administrative software is licensed under a new license, the Delayed Open Source Attribution License (DOSA-1.0) [84]. Although it is not itself an open-source license, the purpose of this Delayed Open Source Attribution License is to provide open access to software for noncommercial use while giving attribution to its original developer, and after a delay of 3 years, forcing the software to fall under the terms of the open source CPAL-1.0 license that preserves the attribution information of this license. This delay is intended to protect the commercial interests of the licensor without compromising on the many benefits of creating open-source products [85].

The backend API is built in Python using Girder’s RESTful API [86] with the CherryPy framework [87]. This software layer provides a set of RESTful endpoints that allow users, applets, activities, items (such as individual questions), and user responses. All user data are stored in a MongoDB database hosted in an Amazon Web Services [88] cloud instance with password-based encryption. Specifically, user response data are encrypted using their own password on the client side so that only managers or reviewers can view their data using an applet password; other sensitive information (name and email) is encrypted on the server side. We have Health Insurance Portability and Accountability Act compliance agreements with Amazon Web Services, Google Cloud Platform [89], and MongoDB Atlas [90], and the software permits installation on an arbitrary backend server (eg, on a university or hospital server and not on any cloud service provider’s servers). For improved performance, MindLogger uses a Redis [91] instance as a temporary storage for data caching. MindLogger uses Firebase Cloud Messaging to send notifications from the backend server to the end user's mobile device. All additional data are consumed from the backend API through the HTTPS requests.

The applets, activities, and items are described using ReproSchema [92], an emerging standard for capturing and harmonizing cognitive, clinical, and behavioral assessments and responses in a provenance-preserving manner. The schema uses JSON for linking data (JSON-LD) [93] as its representation format and captures, as a connected graph of information, the details of the questions, presentation logic on the basis of responses or scheduling, computation of scores, and interface hints for applications such as MindLogger. The schema uses GitHub to maintain versions and provide persistent Uniform Resource Identifiers for applets and activities, supports multilingual applets, and uses World Wide Web Consortium provenance specifications [94] to establish provenance between the response, the responder, and the applet.

#### MindLogger Current Features

**Figure 7:**
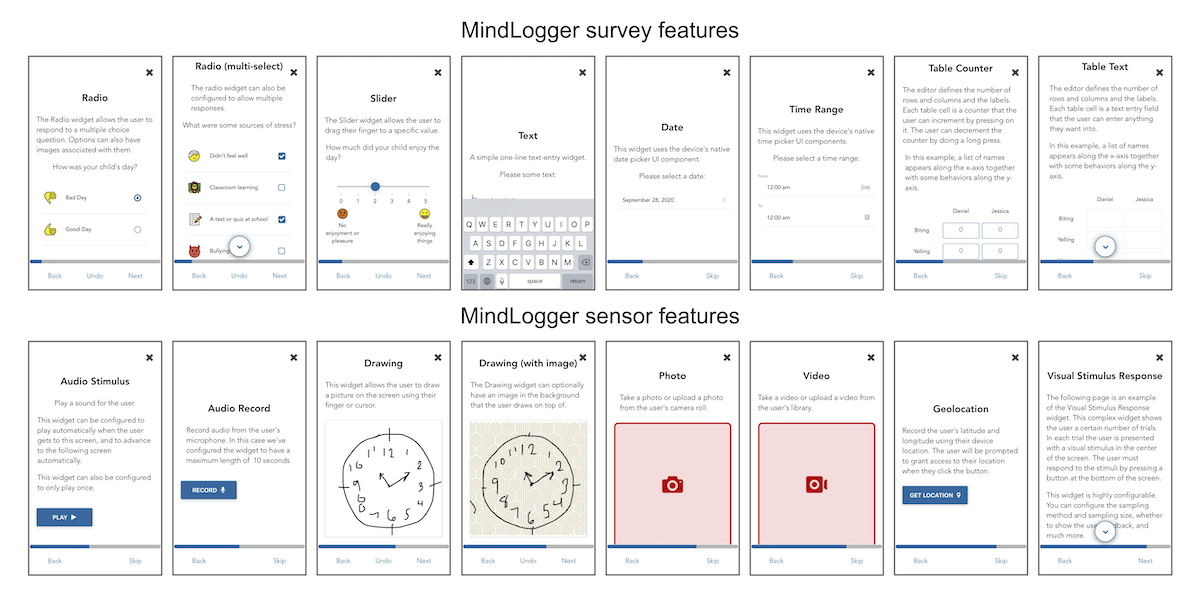
MindLogger screenshots showing survey and sensor features.

We have succeeded in implementing many user- and administrator-facing features (see the MindLogger website [59] for updated information about features, installation, administration, and use). There are an arbitrary number of activities in an applet and an arbitrary number of screens in an activity (with response-based conditional logic directing the sequence of screens). Each screen of a MindLogger applet can display text and a picture or video, play a sound file, and present an interactive component with different possible response options, such as single- or multiple-selection check boxes, image selection, slider bar, text entry, table text or number entry, audio recording, photo or video capture, drawing or tapping on images, or GPS location button. Response delay and timer options are also available for each screen. MindLogger is cross-platform (iOS, Android, and web browser compatibility) and has open-source code for apps and applets, and data are end-to-end encrypted. The browser-based administration panel enables user management, easy creation, and customization of one’s own mobile or web applications without programming or design experience, scheduling of applets and notifications per activity per user or group of users, and visualization and export of data. Figure 7 shows screenshots of the MindLogger mobile phone app features. We have created applets to remotely administer assessments as well as therapies and are currently constructing a public library with over 100 mental health and cognitive assessments that have open licenses for general use.

### Deployment of MindLogger in the Healthy Brain Network Study

**Figure 8:**
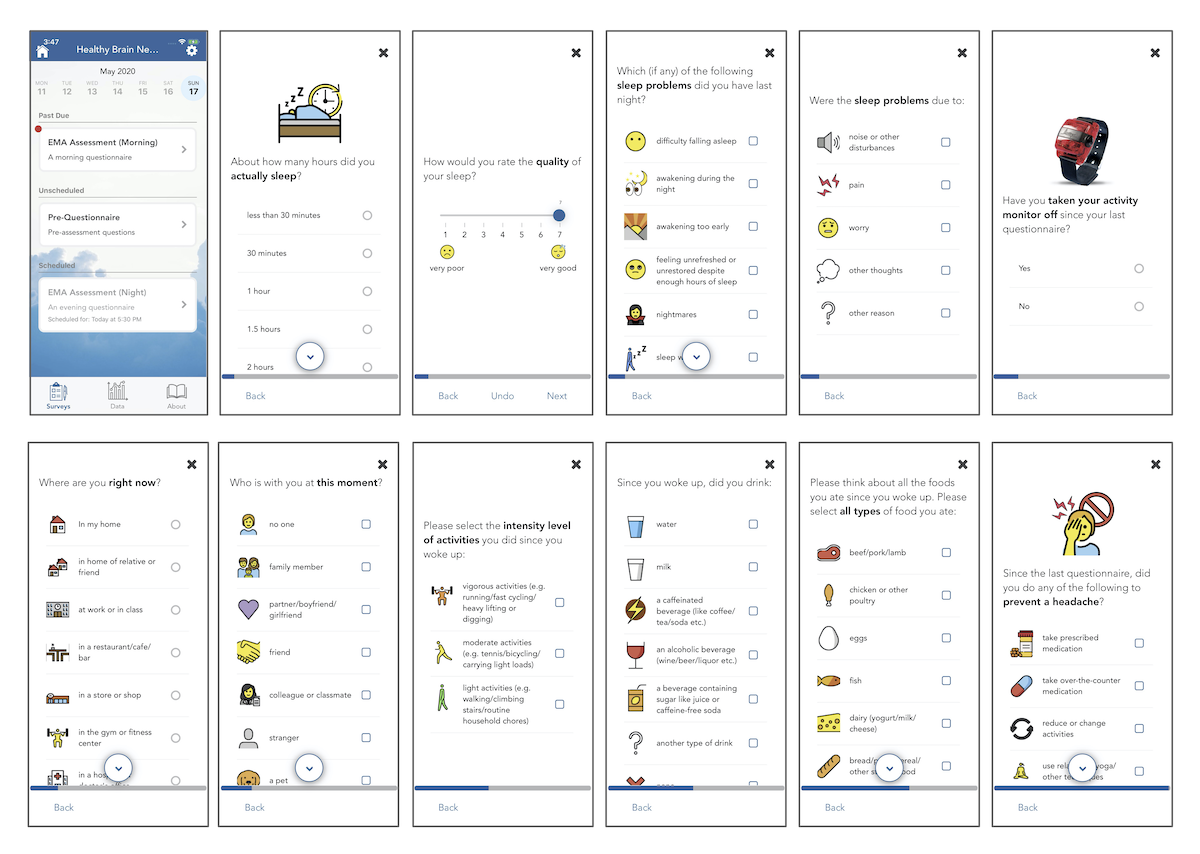
MindLogger National Institute of Mental Health-ecological momentary assessment applet screenshots.

MindLogger’s *NIMH-EMA* applet has been launched as part of the Healthy Brain Network study [54] to a vulnerable transdiagnostic New York City community sample (current n=4315; enrollment rate: 90 per month; >90% have mental health or learning disorders). Participants of the study receive multiple notifications per day on their Android or iOS devices to respond to morning, afternoon, and evening assessments. Figure 8 shows screenshots of the NIMH-EMA applet. We are currently enrolling children and adolescents who are at least 11 years old to use the NIMH-EMA applet as part of the Healthy Brain Network study. This applet is about to be deployed in the NIMH research program as well.

### EDUCATE Study’s Daily and Weekly Diary Applet

Having developed and deployed the NIMH-EMA applet as part of one research study, we were able to rapidly develop and deploy a second assessment applet as part of a different research study on reading disability. Reading disability is the most common learning disability, affecting 10%-15% of school-age children [95]. It causes major functional impairments at all stages of life. A wealth of data documents lifelong disadvantages in educational and occupational attainment. Current evidence-based reading interventions largely rely on services by trained specialists, either in well-resourced classrooms or clinical settings. As such, under-resourced schools (or regions) are often unable to provide reading interventions for their students. The significance of this dilemma is compounded when considering that children of lower socioeconomic status and children with other serious comorbid behavioral health conditions may have more severe or complex reading disability profiles [96-98]. Thus, the children most in need are the least likely to have access to evidence-based treatment.

The EDUCATE study, funded by the National Institute of Child Health and Human Development, is a collaborative effort between researchers at the University of Connecticut and the Child Mind Institute. This clinical trial will examine the effectiveness of an at-home, game-based intervention for reading disorders. Parents of participants in this study are completing daily and weekly assessments in MindLogger, which will allow researchers to assess the home environment and compliance with the intervention protocol throughout the study period. These data are critical for evaluating the impact of this clinical trial.

## Discussion

### Principal Findings

In this paper, we reviewed customizable, mobile experience sampling products for configurable data collection and content delivery, summarized the motivation for and development of a new mobile platform called MindLogger, and described an initial use case that applies a MindLogger version of the NIMH Family Study app as part of the Healthy Brain Network study.

Our review returned an initial set of 392 products, of which 59 appeared to be in current use, had Android and iOS mobile apps, and were capable of scheduling questionnaires and notifications for a group of respondents. Of the 47 companies that responded to our inquiries and did not require a legal agreement, we identified 10 products that satisfied our primary criteria (administrator interface for creating and scheduling recurring, customized questionnaires, where an arbitrary number of users can receive and respond to scheduled notifications on their mobile devices). Four of these products supported end-to-end encryption, 2 enabled restricted access to individual users’ data, 1 provided open-source software, and none provided all 3 of these capabilities. Our review is not exhaustive regarding either the products that could possibly be used for experience sampling or the range of features that these products support. Existing products include an impressive assortment of nonoverlapping features and complement each other in ways that reflect the different niches or markets for which they were intended. The limitations of our search for existing products are as follows: (1) our search queries and Google’s search algorithm may not reflect the optimal search criteria for some relevant products; (2) product websites are sometimes unclear and incomplete and may even misrepresent the capabilities of their products; and (3) some companies did not respond to questions even through their website’s question or support page. Products are also adding features over time, and some companies or organizations offer paid services to build the desired features. Our comparison should be seen as a snapshot of the current state of a subset of features provided by software products intended for experience sampling.

We successfully developed MindLogger, a new platform that meets most of the stated needs of our collaborators around the world, who desire an open source, mobile mental health platform to inform, assess health, acquire data, and administer therapies. We prioritized clinicians’ and researchers’ needs and users’ experience during development, aligned technologies to meet these priorities, and are on track to achieve the full feature set we set out to include. MindLogger has end-to-end encryption, enables restricted access, is open source, and supports a variety of data collection features. One limitation of MindLogger is that it currently does not support passive data collection or interaction with peripheral devices. This was intended to reduce concerns about surveillance in an app whose first use case was for assessing children and adolescents. However, in the future, we intend to support passive monitoring of location and behaviors, and communication with other devices, so long as these are opt-in by the end user and there are clear reminders to the end user concerning what data are collected and how they will be used. Another potential limitation lies in its core strength: by making MindLogger flexible, modular, cross-platform, and configurable to help meet the unforeseen needs of future applet builders and users, the creation of variants of even well-vetted instruments is likely and will necessitate their careful validation. We have recently built a web-based *applet library* for viewing, copying, editing, and sharing applets. Applets in the library are labeled to indicate which have been created or vetted by the Child Mind Institute and which have been contributed by others.

We have demonstrated the flexibility and applicability of the MindLogger platform through the deployment of the NIMH-EMA applet in the Healthy Brain Network, a large-scale, longitudinal, mobile mental health study. The NIMH is about to launch the same applet in its own research program. For future directions in the near term, there are a variety of other mental health–related applets in preparation for deployment and in the planning stages as described below.

### MindLogger Applets in Preparation for Deployment

We are currently refining and testing MindLogger applets to assess and administer interventions targeting specific subgroups of youth with particular mental health and learning disorders. Although some of these applets support specific collaborators’ research, others are for broader use (listed below, with video screencast demonstrations on the MindLogger website [59]).

#### Pediatric Screener Mental Health Screening Applet

Integrating primary care and mental health has been associated with improved patient outcomes [99]; therefore, mental health screening in pediatric clinics could lead to earlier diagnosis and improved outcomes for patients. The Hearst Foundations supported the development of a pediatric screener tool using MindLogger. This tool will administer assessments to children or their parents, for children receiving a wellness checkup at their pediatric clinic, and alert their physician if a child shows signs of a mental health disorder. We have built the applet and will pilot it at the Richmond University Medical Center in Staten Island, New York. The initial screening questionnaire assesses internalizing and externalizing symptoms, issues of attention and hyperactivity, depression and suicidal ideation, disordered eating behavior, and experiences of bullying. It also collects demographic information about the child and parent. Children with a clinically significant level of symptoms were prompted to complete additional questionnaires to collect more detailed information. A similar questionnaire was piloted in several New York City–based pediatric settings and found it to be an effective tool for identifying children at risk of a serious mental health disorder. The MindLogger platform will create a much more streamlined process and user-friendly experience, increasing the probability of adoption by more pediatric practices and clinics.

#### Dialectical Behavior Therapy Applet

**Figure 9:**
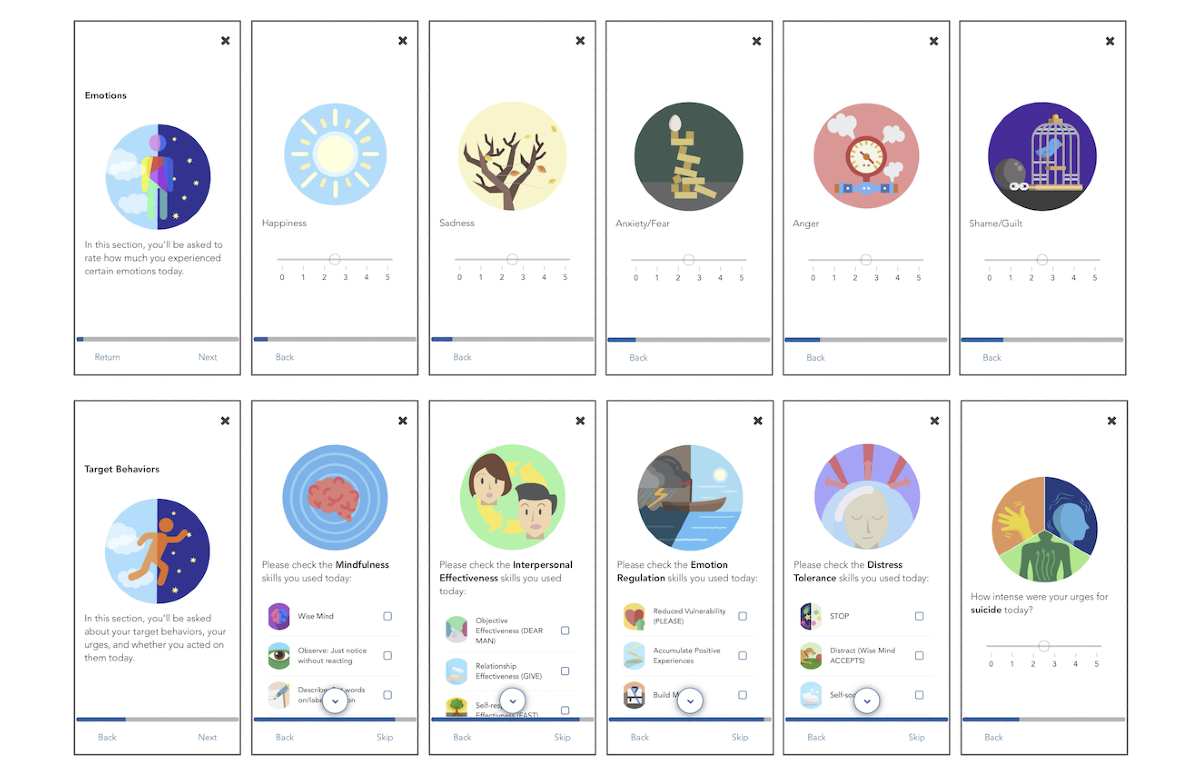
MindLogger Dialectical Behavior Therapy applet screenshots.

The Dialectical Behavior Therapy (DBT) Diary Card is a digitized version of the diary card used in evidence-based DBT programs. This tool is a daily tracker of mood, targeting behavioral urges and specific behaviors, and the use of coping skills to manage these emotions, urges, and behaviors. Our DBT applet (Figure 9) will enable therapists to create a digital DBT diary card with a patient to include specific treatment targets for that individual. This will allow patients to set notification reminders to complete the diary card on a daily basis. The patient’s device will automatically update with the new targets and schedule, allowing the user to progress in the therapy without having to change the way the data are retrieved. Both patients and therapists will have access to the diary card data to guide treatment planning and sessions.

#### TokenLogger Behavior Intervention Applet

We are refining a behavior intervention applet called *TokenLogger* that can be customized to retrospectively and prospectively track specific behaviors and help promote and reinforce desirable behaviors while reducing or extinguishing undesirable behaviors. It will also help track the frequency, duration, and severity of target behaviors to inform modifications in behavioral treatment plans and assess progress toward outcomes and goals. The TokenLogger applet will be evaluated by clinical experts at the Child Mind Institute and piloted with patients at the Child Mind Medical Practice, to better understand the timing, duration, and frequency of undesirable behaviors, and to test the efficacy of this digital rendition of behavior modification therapy.

### Planned MindLogger Applets

We intend to replace all of the Child Mind Institute’s and the Child Mind Medical Practice’s pencil-and-paper assessments with MindLogger applets and, where appropriate, create and test digital renditions of therapies as MindLogger applets, and make these universally accessible as part of a web-based public library of MindLogger applets for anyone to use, modify, and translate. In addition, there are a few applets in the planning stages.

#### Diagnostic Screening Applet for Kiddie Schedule for Affective Disorders and Schizophrenia

We are currently developing a MindLogger applet with part of the Composite International Diagnostic Interview Screener that has been incorporated into the NIMH version of the epidemiologic version of the Kiddie Schedule for Affective Disorders and Schizophrenia. This screener is being tested for parents and self-administration in order to streamline the process as we transition to more automated approaches for large-scale studies of youth.

#### Taction Exposure Therapy Applet

We created a prototype iOS and Android mobile app called *Taction* that is a simple exposure therapy game for children who have obsessive-compulsive disorder or anxiety-related issues. The app rewards users for tapping on images that heighten their anxiety, potentially helping them progress in their treatment between exposure therapy sessions. Once incorporated into MindLogger, the Taction applet will be evaluated at the Child Mind Institute and piloted with patients at the Child Mind Medical Practice to assess the relative efficacy of a digital rendition of exposure therapy.

### NIMH Cognitive Task Battery

In the same manner as we want to enable anyone to create, configure, and administer their own mobile questionnaires, we also want to enable anyone to do the same for different types of cognitive assessments. In this spirit, we created a Flanker task applet that is thoroughly configurable (presentation and timing of a fixation target, stimulus, and feedback), and we are collaborating with the NIMH to create a small battery of cognitive tasks for research in modeling behaviors.

## Appendix

Multimedia Appendix 1 Protocol for product search.

## Abbreviations

API: application programming interface
DBT: Dialectical Behavior Therapy
EMA: ecological momentary assessment
MATTER: Mind-Assisting Technologies for Therapy, Education, and Research
MRI: magnetic resonance imaging
NIMH: National Institute of Mental Health
