# Supplementary material for: Remote Digital Psychiatry for Mobile Mental Health Assessment and Therapy: MindLogger Platform Development Study
Source: J Med Internet Res. 2021 Nov 11;23(11):e22369. doi: 10.2196/22369 (PMC8663601; doi:10.2196/22369)
Supplement: Multimedia Appendix 1 [file jmir_v23i11e22369_app1.docx]

# Supplement to “Remote Digital Psychiatry: MindLogger for Mobile Mental Health Assessment and Therapy”:

# Protocol for Table 1

## Review criteria for customizable data collection tools

In this Supplement, we outline the protocol we followed to gather information about products that appear to have functionality like MindLogger. Many products’ websites were unclear about pricing, privacy, and encryption and deletion of data. We did not enclose any search queries with quotation marks unless explicitly indicated.

### **Search strategy**

1. Collect the first 20 Google search results (no ads) for each of three queries:
   1. Query #1: “digital electronic data capture systems”
   2. Query #2: “alternative to qualtrics”
   3. Query #3: “mobile phone software sensor data collection”

Results from prior queries are not included among the results of subsequent queries.

1. Search for websites for products identified by our researchers and clinicians (only for products that were not among those found by the above queries).
2. Follow product leads in the search results. For example, if a website says “Top ten apps for...” then we conduct additional online searches for websites for each of the 10 apps that fit our inclusion criteria (below). We rely on official product websites, GitHub repositories of the creators of the products, or official apps on Apple’s App Store or Google Play. If the name of the project is too generic, we add the name of the company to the search or add quotation marks around the project name.
3. Include only those products that are currently in use.
   1. To be currently in use, product must have an active website, and any of the following:
      1. Website contains information for obtaining the product (“Download”, “Book a demo”, “Contact us to get a quote”, or a similar phrase)
      2. Last release date was within the last two years ( to October 13, 2020), and the most recent date from any of these sources:
      3. Release notes (“release” or “version”) in product site’s news/blog
      4. Release information in a GitHub open source repository
      5. App Store “version info” or Google Play “last updated”
      6. Google search: “[project name] last release”
      7. Latest commit date in open source repository
4. Include only those products:
   1. whose content is customizable (e.g., can build questions and add images or audio) and/or
   2. that allow users to collect sensor data (accelerometer, GPS, etc.)
5. Include only those products that have a mobile component (Android and/or iOS).
   1. Search website directly for “app” and “application” [Notes 1-5]
   2. Search website using Google: “*site:<domain name> <keyword>*”, where <keyword> is any of “app”, “android”, “ios”, “store”, “google play store”, “app store”, “mobile”, “native”; *example: “site:<mindlogger.org> <app>”* [Notes 1-5]
   3. Search website for links to Apple’s App Store or Google Play.
   4. Search Google: *“<product name> <keyword>”* using above keywords. [Note 6]

### **Additional information about each product**

To determine whether a product includes additional features of interest, we addressed the following questions in our search strategy:

1. Can a person using the product to add, for example, a survey question to an app include any of the following, or can a person use the product to respond to a question with any of the following?:
   1. Text
   2. Image
   3. Audio
   4. Video
   5. GPS
   6. Notifications / reminders / alerts
2. Does the product include access to additional sensors (e.g., accelerometer, gyroscope,...)?
3. Does the product include a license?
   1. Website has license information
   2. Website has terms of use that mentions a license
   3. GitHub repository, if available, includes a license file, or mentions a license in its README file (ignored if multiple repositories with different licenses)
   4. Google search “[project name] license”
4. Does the product encrypt data?
   1. Google search: “[project name] encrypted ssl tls”
5. Does the product have a privacy policy? [Notes 7-8]
   1. Google “[project name] privacy policy”
6. Can a user delete their data?
   1. Google search: “[project name] delete data”
      1. Satisfied if a software feature, not if a company deletes data on request
      2. Satisfied if data automatically deletes after a prescribed period of time
7. Do you have to pay money to use the product?
   1. Search product website for cost or pricing
   2. If pricing information not available, search website for “request demo”, “contact”, or other relevant wording, or “not available”, as appropriate
   3. Try to determine if product falls into one of these categories:
      - 1. Free
        2. Payment/in-app purchase/subscription (may also include a free version)
        3. Unknown / request demo

## Notes

[1] “Mobile” specifically refers to “mobile app” or “for building mobile apps”; not, for example, “mobile data collection”.

[2] “Android application”, “Native mobile apps”, “Available on Apple (iOS) and Android devices”, and “supports ios and android devices” are all positive indicators.

[3] Phrases such as “works on desktop and mobile” and “compatible with android and ios devices” are not direct indicators, so follow any leads/links.

[4] Check in product’s list of software requirements.

[5] The product itself must be an app, and not for building apps, and search results need to clearly state that the product is a mobile/native app, not a web app that works on iOS/Android.

[6] Sometimes apps are in the app stores but not mentioned on the product’s website.

[7] The privacy statement can be called something else, but should contain a phrase like “in this privacy policy”.

[8] Check that the statement about privacy is about the app or the whole company, not specifically the product’s website, (e.g. “products or services, including our website”).
